# Point-of-care nano zinc oxide carbon paste sensor for non-invasive determination of clozapine in saliva samples

**DOI:** 10.1186/s13065-025-01671-3

**Published:** 2025-11-15

**Authors:** Mariam O. Abd el-Aziz, Amr M. Bekhet, Hany H. Monir, Sameh E. Younis, M. Nebsen, Ahmed H. Nadim

**Affiliations:** 1 Department of Pharmaceutical Chemistry, Faculty of Pharmacy, Pharos University in Alexandria;, Canal El Mahmoudia Street, Beside Green Plaza Complex 21648, Alexandria, Egypt; 2 Pharmaceutical Analytical Chemistry Department, Faculty of Pharmacy, Cairo University, Kasr El-Aini Street, Cairo ET-11562, Egypt

**Keywords:** Ag-doped ZnO nanoparticles, Clozapine, Electrochemical sensor, Point-of-care test, Saliva, Therapeutic drug monitoring

## Abstract

Clozapine (CLZ); an atypical antipsychotic drug, is well known to have a significant role in managing schizophrenic patients with substance use disorder (SUD). Unfortunately, many patients are deprived of CLZ benefits due to its limited prescription. This is based upon concerns regarding the critical side effects of CLZ in case of overdosing especially, with the lack of accessible therapeutic drug monitoring (TDM) tools. In this contribution, a simple, accurate and sensitive electrochemical method is proposed for CLZ assay in human saliva. Unlike previously reported methods for TDM of CLZ that depends on invasive matrices as plasma and urine, this method employs electrochemical approaches in exploring human saliva as a patient-friendly alternative for assessing CLZ. The proposed method employs differential pulse voltammetry (DPV) with a sensitive and selective Ag-doped ZnO nanoparticles based carbon paste electrode (CPE). The adopted electrochemical sensor has not been previously reported for CLZ determination, despite it offers enhanced sensitivity together with simple synthesis. The synthesized nanoparticles were characterized through Fourier-transform infrared spectroscopy (FTIR), scanning electron microscopy (SEM) and energy dispersive X-ray spectroscopy (EDX). The developed sensor was optimized and validated as per FDA guidelines of bioanalytical methods. The linear range in saliva was 0.31–3.67 µmol/L and the lower limit of quantitation (LLOQ) was 0.31 µmol/L. The high reliability and applicability of the suggested method has strong potential to be integrated in a point-of-care testing (POCT) device to introduce more accessible TDM that enables smooth TDM of CLZ. Therefore, it opens pathways for broader and safe use of CLZ.

**Graphical abstract:**

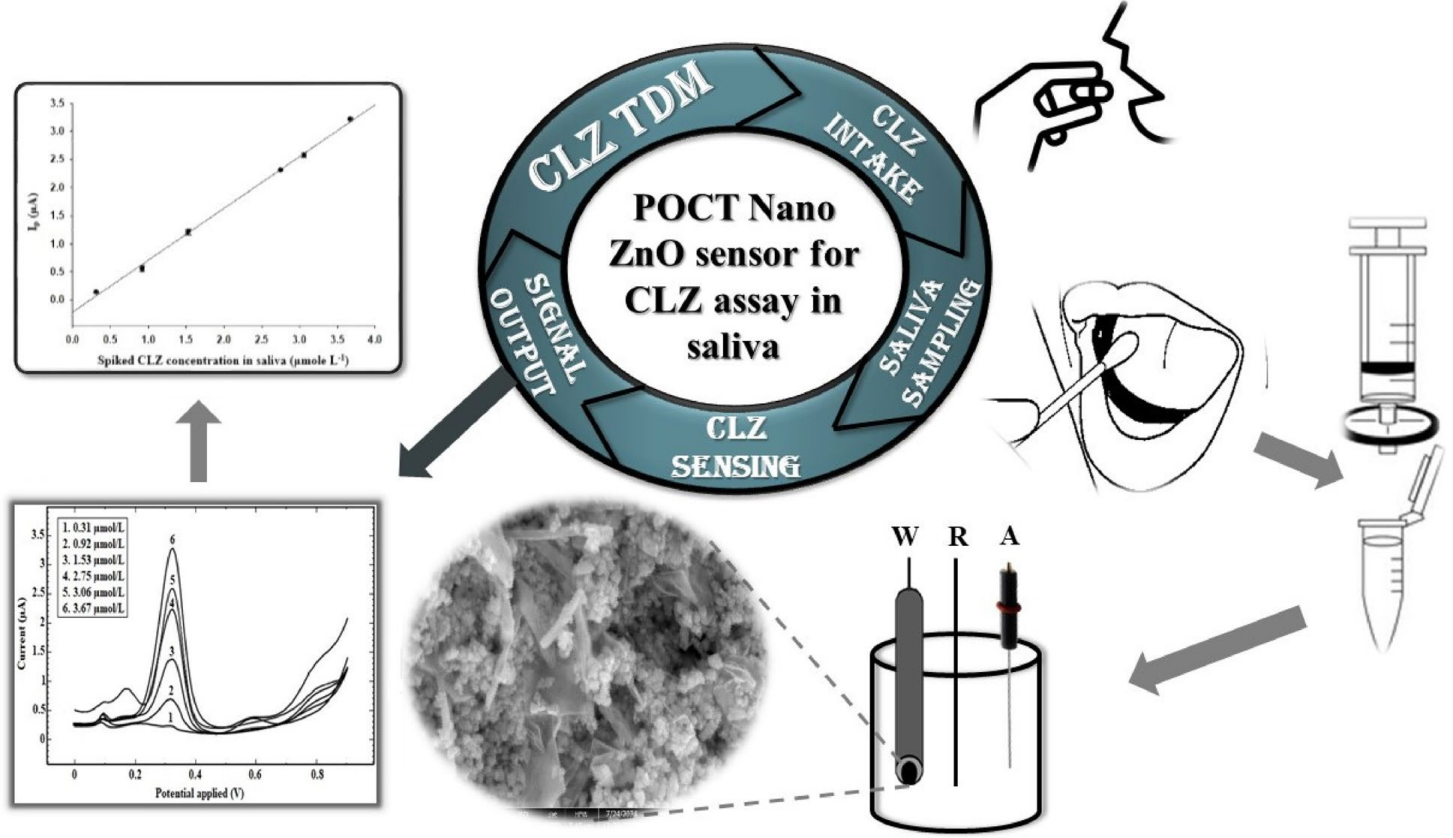

## Introduction

Substance use disorder (SUD) takes place when psychoactive substances are being administered in overdoses and over long periods. It is commonly referred to as substance abuse. As a result, brain damage occurs followed by the development of serious mental disorders, including schizophrenia, bipolar depression and anxiety. Notably, this may boost the rewarding effects of abused substances leading to inability to resist the urge to use them. SUD and co-occurring mental disorders are believed to be common interrelated comorbidities [1]. Each one has the potential to precipitate the other interchangeably. Such comorbidities lead to serious health as well as social consequences in terms of psychotic and depressive symptoms that definitely worsens patients’ life quality. At this stage, appropriate therapeutic interventions have to be considered immediately to treat both disorders simultaneously. Recent studies unleashed clozapine (CLZ) as a drug of choice for treating schizophrenic patients with comorbid SUD [2].

CLZ is chemically named as 8-chloro-11-(4-methyl-1-piperazinyl)-5 H-dibenzo [1, 4] diazepine [3]. It is a superior medication that belongs to the second generation of atypical antipsychotics. Its superiority is due to its action through various receptors; it is an antagonist of serotonin, dopamine, alpha adrenergic, histaminic and muscarinic receptors. Thus, it plays a crucial role in enhancing negative, positive and cognitive symptoms without inducing any extrapyramidal side effects [4]. Owing to this multimodal mechanism of action, CLZ is highly recommended in treating resistant schizophrenic patients especially those with suicidal symptoms. Moreover, it has an off-label use in controlling SUD [5]. Even though CLZ has a proven high efficacy in managing schizophrenic patients with co-occurring SUD, health care professionals have not yet fully exploited it as a first line therapy. This is referred to its life threatening side effects encountered with high doses. The frequent observed symptoms of CLZ overdosing are agranulocytosis, seizures, myocarditis and coma [6]. Thus, it is necessary to regularly monitor biological CLZ levels to ensure attaining therapeutic rather than toxic effects. A low CLZ dose of 150–300 mg/day could be administered with lower side effects. A standard dose of 300–600 mg CLZ/day could be optimal in improving symptoms but is associated with more side effects. CLZ doses above 900 mg/day could be toxic [7].

Therapeutic drug monitoring (TDM) of CLZ is critical and has to be adopted on regular basis to check CLZ levels in patients throughout their treatment period [8]. A rigorous literature survey revealed many analytical methods for CLZ determination in different biological fluids. These methods include: Spectrophotometry [9], RP-HPLC [10–13], LC/MS [14] and GC/MS [15, 16]. In spite of the accuracy of these methods, they operate in sophisticated and time consuming setups. In addition, they require highly trained personnel and high budget. Thus, they have limitations regarding regular TDM. On the contrary, electroanalytical methods are reliable, compatible with miniaturization, simple (i.e.; they require almost neither pretreatment steps nor highly trained personnel), cost effective together with being ecofriendly. They also possess elevated selectivity based on the characteristic oxidation/reduction behavior of each molecule. Moreover, nanomaterials integration in electrochemical sensor’s fabrication boosts the sensitivity of electroanalytical methods to extremely high levels. Among of the previously developed nanomaterials-based modified electrodes for antipsychotics determination; Multi-walled carbon nanotubes (MWCNTs) for sulpiride, metal nanomaterials for chlorpromazine, molecularly imprinted polymers and nanoparticles for midazolam and composite sensors for vortioxetine [17]. Thus, they are proposed as valuable alternative which is more suitable for assaying not only CLZ, but also many more antipsychotics [18]. Reviewing the literature revealed some electroanalytical methods developed for assaying CLZ in biological fluids, mainly blood, such as cyclic voltammetry [19], linear sweep voltammetry [20], square wave voltammetry [19, 21], differential pulse voltammetry [19, 22–24] and micro fabricated electrochemical lab-on-chip device [25]. However, relying on plasma and blood samples in TDM is very tedious. Blood samples require multiple prerequisite treatment steps to be ready for analysis. In addition, it represents unpleasant sampling route to overwhelmed schizophrenic addicts. Moreover, CLZ is approximately 95% bound to plasma proteins [26]. Therefore, health care professionals would struggle to conduct it regularly.

On the other hand, saliva has been suggested to be an appropriate substitute to plasma for TDM purposes [27]. This is empowered by the accurate reflection of CLZ serum levels through its concentrations in saliva [27–29]. However, only few studies presented CLZ assay in saliva; HPLC [12, 13], UPLC [30] and GC/MS [15]. Up till now, only one electro-based sensor was developed for CLZ determination in rat saliva [27].

Finding a reliable, sensitive, simple and patient friendly analytical method is crucial to ensure maintaining CLZ at its safe therapeutic range (1.68–2.81 µmol/L in saliva) [30]. Regarding electrochemical drug analysis, pulse techniques are impressively gaining superiority, due to their ability to effectively minimize background noise, that results in excellent sensitivity [31]. They include normal pulse voltammetry (NPV), differential pulse voltammetry (DPV) and square wave voltammetry (SWV). DPV technique operates by applying amplitude potential pulses, in increasing manner, to permit oxidation/reduction processes at electrode’s surface. Then, the resulting current is measured. Its major advantage is having low capacitive current which contributes to its sensitivity compared to other voltammetric techniques [32, 33]. Thus, it is capable of assaying trace levels of drugs in various complex matrices. Carbon paste electrodes (CPE) are on top of the most commonly adopted electrodes in electroanalytical methods. This is referred to having low residual current, broad potential range, stability, economic and facile fabrication together with continuous surface regeneration ability. Furthermore, it possesses a promising potential in hosting the technology of nanoparticles for drugs assay. In addition, doping of nanoparticles with metals enhances their sensitivity and performance as electrochemical sensors for tracking trace levels of drugs in biological samples [34, 35]. In this regard, Ag-doped ZnO nanoparticles (Ag-doped ZnO NPs) can be used for modification of CPE. This allows unique electrical conductivity, biocompatibility, additional chemical stability over that of CPE itself, larger surface area, powerful ionic bonding that contributes to their superior electro-catalytic activity [36].

The current research is principally targeted to develop an electroanalytical method that utilizes a sensitive electrochemical sensor for the therapeutic monitoring of CLZ in human saliva. A DPV method was proposed together with developing Ag-doped ZnO NPs based CPE. Optimization and validation of the developed bioanalytical method were conducted according to FDA guidelines. In addition, full characterization of the synthesized nanoparticles was accomplished using Fourier-transform Infrared spectroscopy (FTIR), scanning electron microscopy (SEM), energy dispersive X-ray spectroscopy (EDX), and electrochemical impedance spectroscopy (EIS) techniques.

The proposed method aimed to present a simple, cost effective, non-invasive and POCT-compatible approach to facilitate routine TDM of CLZ in the management of schizophrenic patients with comorbid SUD.

## Materials and methods

### Instrumentation

The electrochemical experiments including electrochemical impedance spectroscopy (EIS) were conducted on potentiostat/galvanostat PGSTAT204 device (Metrohm, Netherlands) operated with NOVA 1.11.2 software. The electrochemical cell was composed of; Ag/AgCl as a reference electrode, platinum wire as a counter electrode and Ag-doped ZnO NPs modified CPE as a working electrode. pH adjustments were carried out on Jenway pH-meter 3310 (Dunmow, Essex, United Kingdom). The characterization of the synthesized nanoparticles was carried out using FTIR (Perkin Elmer, USA) with spectra recorded over a range of 450–4000 cm^− 1^ and scanning electron microscopy SEM Quanta 250 FEG (FEI, Netherlands) with magnification power of 120000x.

### Chemicals and reagents

Clozapine (CLZ) of 99.9% purity was obtained as a gift from Apex Pharma. Co., Cairo, Egypt. Analytical grade zinc acetate, silver nitrate, cobalt chloride, graphite powder, paraffin oil, phosphoric acid, acetic acid, boric acid, NaOH, polyethylene glycol (PEG-400) and methanol were purchased from El-Nasr chemical Co., Alexandria, Egypt, and the redox probe potassium ferrocyanide (K_4_[Fe(CN)_6_]) / potassium ferricyanide (K_3_[Fe(CN)_6_]) was provided by Sigma-Aldrich, Darmstadt, Germany. Britton-Robinson buffer (BRB); 0.04 mol/L, used for pH adjustments was prepared by mixing equal volumes of 0.04 mol/L phosphoric, acetic and boric acid. Then, appropriate volumes of 0.2 mol/L NaOH were added to adjust the pH over the range (2.0–10.0) [37]. Blank human saliva was obtained from healthy volunteers. Freshly prepared deionized water was used in all experiments.

### Fabrication of the electrochemical sensor

#### Synthesis of ZnO NPs and metal-doped ZnO NPs

The un-doped ZnO NPs were synthesized following the standard co-precipitation method [38]. Fifty milliliters of 0.1 mol/L zinc acetate were prepared in deionized water as a precursor solution. It was heated at 60 °C. Then, 0.8 mol/L NaOH and PEG-400 (95:5 v/v) mixture solution was gradually added to the heated zinc acetate solution. Under magnetic stirring, this solution was agitated at 60 °C for 4.0 h. The produced white precipitate was then separated by centrifugation at 4000 rpm for 10 min. It was purified by applying three cycles of washing with alcohol and deionized water, accompanied by 10-minutes centrifugation at 4000 rpm for each cycle. The purified precipitate was then dried inside a porcelain dish by hot air oven at 120 °C for 4.0 h.

Both Co-doped ZnO NPs and Ag-doped ZnO NPs were prepared by applying the same procedure. But, 0.1 mol/L of either cobalt chloride or silver nitrate solution was added to the zinc acetate precursor solution, respectively, and colored precipitates were obtained using the same procedure as mentioned for ZnO NPs.

#### Construction of Ag-doped ZnO NPs modified CPE

The modified CPE was constructed by mixing 90 mg of graphite powder with 10 mg of Ag-doped ZnO NPs using 20 µL of paraffin oil as a binder, till a homogenous paste was obtained. Then, this paste was packed, pressed into the cavity of the Teflon piston holder of the working electrode and then smoothed. The surface of the assembled working electrode had to be regenerated prior to each run by polishing it against filter paper.

###  Preparation of stock and working standard solutions

A stock standard solution of CLZ (300 µmol/L) was prepared in methanol. Subsequent dilution with BR buffer (pH 6.0) was carried out to obtain working standard solution (30 µmol/L). CLZ standard solutions had to be refrigerated throughout the whole study.

### Construction of calibration curve in saliva

#### Operational conditions of electrochemical measurements and study of scan rate effect

Preliminary experiments were held to realize the optimum electrochemical conditions for CLZ assay, by adopting differential pulse voltammetry (DPV) and cyclic voltammetry (CV) techniques. DPV voltammograms were recorded at room temperature over a scanning potential ranged from 0.00 to 900 mV. The electrochemical conditions; modulation amplitude, modulation time, interval time, step potential and scan rate, were adjusted at 75 mV, 25 ms, 500 ms, 8.0 mV and 16 mV s^− 1^, respectively. CV voltammograms were also recorded at room temperature over a scanning potential range of -100 to 700 mV vs. Ag/AgCl reference electrode and at a scan rate that extended from 10 to 125 mV s^− 1^.

####  Preparation of human saliva samples

Pooled saliva samples were obtained from ten adult healthy volunteers without any history of medical conditions or drugs intake prior to samples collection. The samples were collected following a previously published technique [39]. First, saliva was allowed to pile up in a cotton swab by placing it in volunteer’s oral cavity for almost 10 min. Then, the cotton swab was transferred to a syringe with a syringe filter fitted at its open end. Finally, the saliva was expelled into a sterile container by squeezing the cotton swab towards the syringe filter through which samples purification was accomplished.

#### Analysis of CLZ in spiked human saliva samples

One-mL aliquots of saliva samples were transferred to series of 25-mL volumetric flasks. Each saliva sample was spiked with CLZ in the concentration range of 0.31–3.67 µmol/L, and completed to mark with BR buffer (pH 6.0). The samples were prepared in triplicates. Under the optimized electrochemical conditions, DPV technique was applied using Ag-doped ZnO NPs modified CPE over a scanning potential range of 0.00–900 mV. Then, the peak height (I_p_) corresponding to each CLZ concentration was recorded. The calibration curve was then constructed by plotting the obtained I_p_ in correspondence to the relative CLZ salivary concentration.

### Preparation of quality controls (QCs)

In accordance with FDA guidelines for validation of bioanalytical methods [40], QCs samples were prepared by spiking blank saliva samples with various aliquots of CLZ at five concentration levels reflecting lower limit of quantitation (LLOQ), low quality control (LQC), mid quality control (MQC), high quality control (MQC) and upper limit of quantitation (ULOQ) (0.31, 0.92, 1.53, 2.75 and 3.67 µmol/L saliva, respectively).

##  Results and discussion

### Study of the effect of ZnO NPs doping on CLZ sensing

**Fig. 1:**
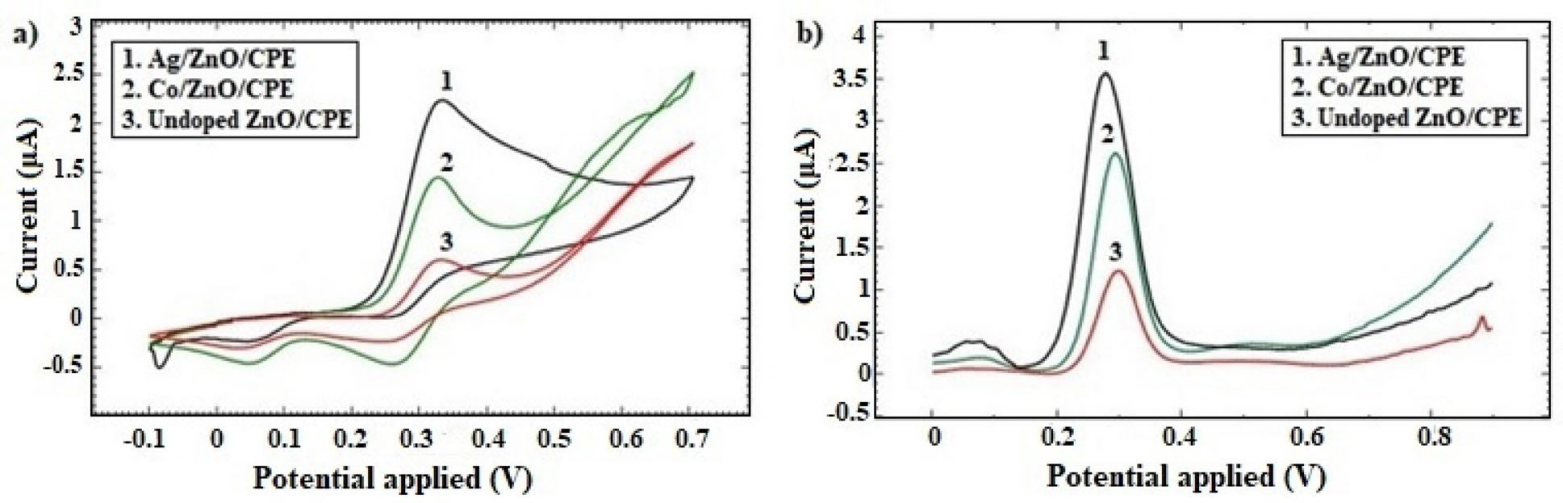
CV **a** and DPV **b** voltammograms of 2.45 mmol/L CLZ in KCl solution using modified CPE manufactured in different approaches

For the purpose of fabricating a sensitive and selective CPE sensor, various exploratory studies were carried out. Both CV and DPV techniques were applied on 2.45 mmol/L of CLZ in KCl supporting electrolyte solution to unleash the optimum composition of the CPE sensor for quantitative determination of CLZ. The optimum dopant type was decided by studying the voltammetric behavior of CLZ at the surface of CPE modified with un-doped ZnO NPs, Co-doped ZnO NPs and Ag-doped ZnO NPs. CV voltammograms (Fig. 1-a) revealed that the highest anodic peak current was obtained with Ag-doped ZnO NPs modified CPE at a potential of + 327 mV. It achieved I_p_ of 1.77 µA. On the other hand, 0.96 and 0.40 µA were obtained with Co-doped and un-doped ZnO NPs modified CPE, respectively. In addition, peak-to-peak separation potential (ΔE_p_) decreased from 65 mV at the un-doped ZnO NPs modified CPE to 55 mV at the Ag-doped ZnO NPs modified CPE, suggesting that Ag doping enhanced charge transfer kinetics at the electrode’s surface. Moreover, DPV voltammograms (Fig. 1-b), showed I_p_ values of 3.42, 2.47 and 1.15 µA for Ag-doped, Co-doped and un-doped ZnO NPs modified CPE, respectively; at 277 mV. Thus, Ag metal as a dopant for ZnO NPs crystals offered better electrocatalytic activity and more feasible oxidation process of CLZ.

### Synthesis and characterization of Ag-doped ZnO NPs

The co-precipitation technique was adopted for synthesis of Ag-doped ZnO NPs. It has notable advantages over other synthesis methods, as it is facile, inexpensive, does not require special atmospheric conditions and ensures uniform distribution of nanoparticles which contributes to enhanced electrochemical performance of the sensor [41]. So that, it is considered a highly convenient technique for obtaining high quality nanoparticles.

#### Surface characterization

**Fig. 2:**
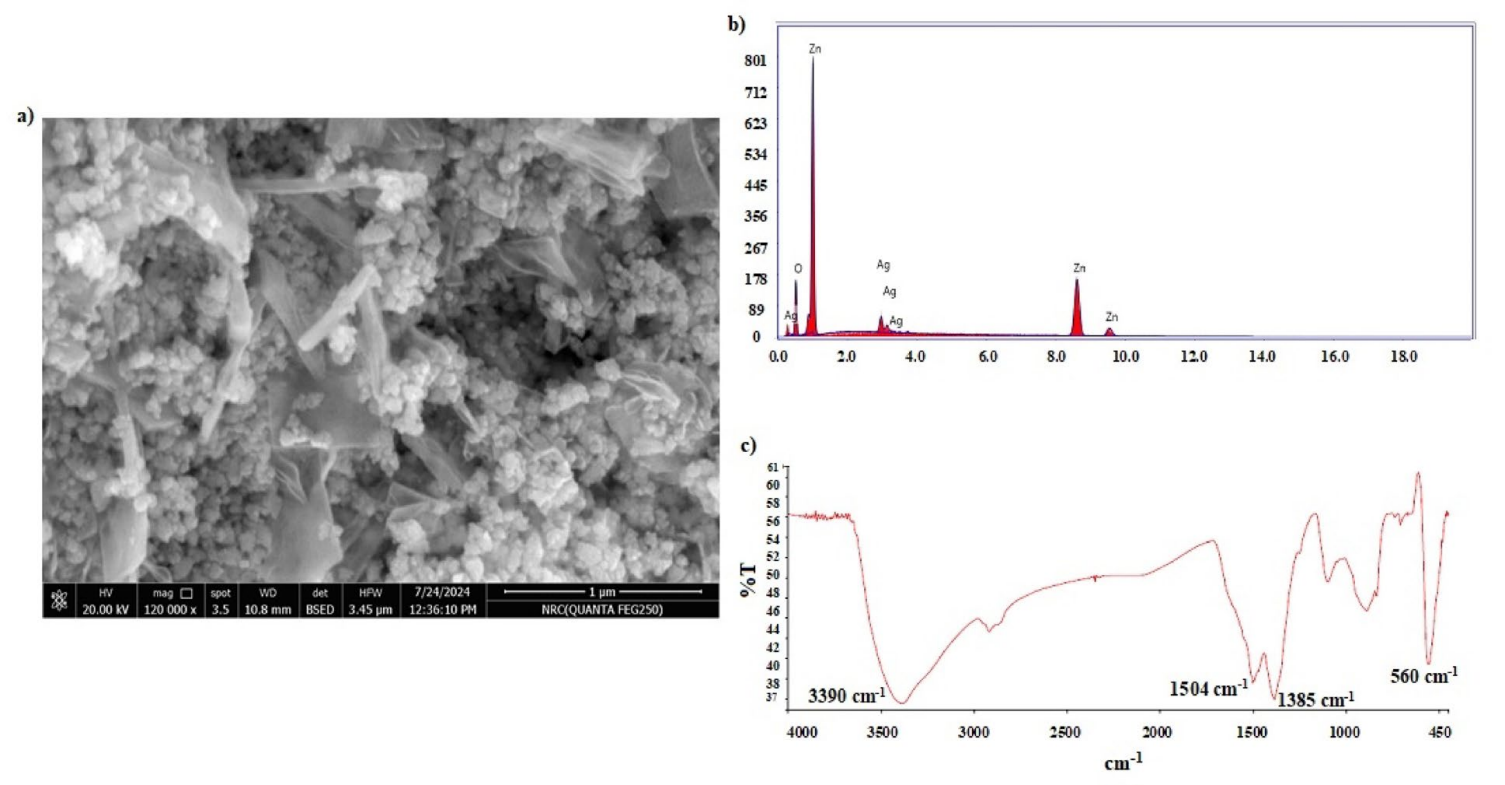
**a** SEM image of Ag-doped ZnO NPs (Magnification power of 120,000x), **b** EDX spectrum of Ag-doped ZnO NPs sample and **c** FTIR spectra of Ag-doped ZnO NPs

The synthesized Ag-doped ZnO NPs were evaluated through various characterization techniques to demonstrate its morphological as well as structural characteristics. SEM was adopted to examine the shape and morphology, EDX for determining the elemental composition, and FTIR spectroscopy to reveal the structure of the synthesized nanoparticles. SEM image of Ag doped-ZnO NPs sample was captured as shown in Fig. 2-a, spherical nanoparticles agglomerated structure of Ag-doped ZnO NPs appeared in SEM images. To confirm the elemental composition of the synthesized NPs; EDX was applied. Its spectrum showed distinctive resolution peaks representing Zn, O, and Ag (Fig. 2-b). No other peaks were observed, declaring that the sample is free from residual impurities like carbon element. The calculated atomic% of the elements of composition was 2.40%, 48.39% and 49.21% for Ag, Zn, and O, respectively. Thus, the elemental ratio of Zn to O was nearly 1: 1. This indicated the presence of enough oxygen vacancies in the sample, which explained the observed porosity of the ZnO NPs lattice. Additionally, the successful doping of Ag metal into ZnO NPs lattice was emphasized by recording its FTIR spectra over the range of 450–4000 cm^− 1^. A significant absorption band appeared at 560 cm^− 1^ representing the stretching vibration mode of ZnO-Ag NPs (Fig. 2-c). Additional peaks appeared at 1385 and 1504 cm^− 1^ referring to the acetate moiety present on the surface of ZnO. Moreover, a broad band was observed at 3390 cm^− 1^ indicating the presence of (OH) groups of water molecules that were thought to be absorbed on the surface of ZnO NPs [42]. As a conclusion, all the obtained data from the employed characterization techniques verified that Ag-doped ZnO NPs has been synthesized effectively.

#### Electrochemical characterization

**Fig. 3:**
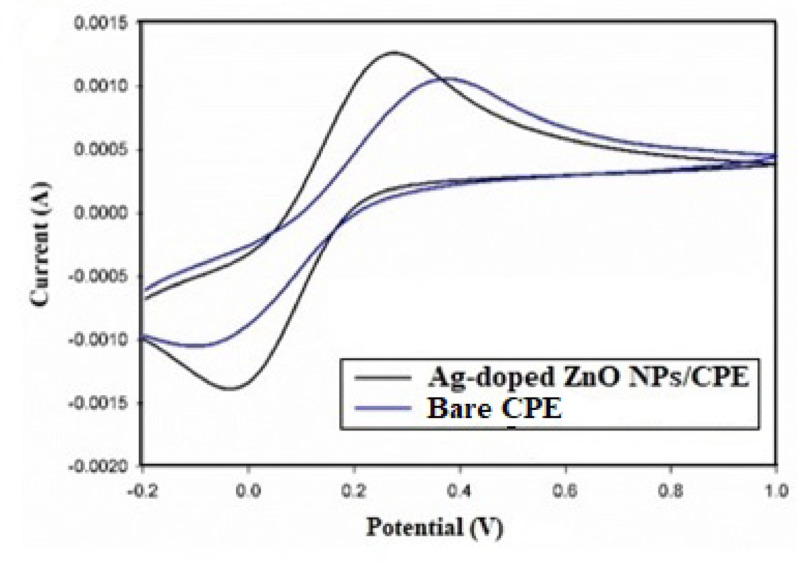
CV voltammograms of bare CPE ( ) and Ag-doped electrode ( ) in 0.1 mol/L KCl solution containing 10 mmol/L (K_4_[Fe(CN)_6_]) / (K_3_[Fe(CN)_6_]) redox couple

**Fig. 4:**
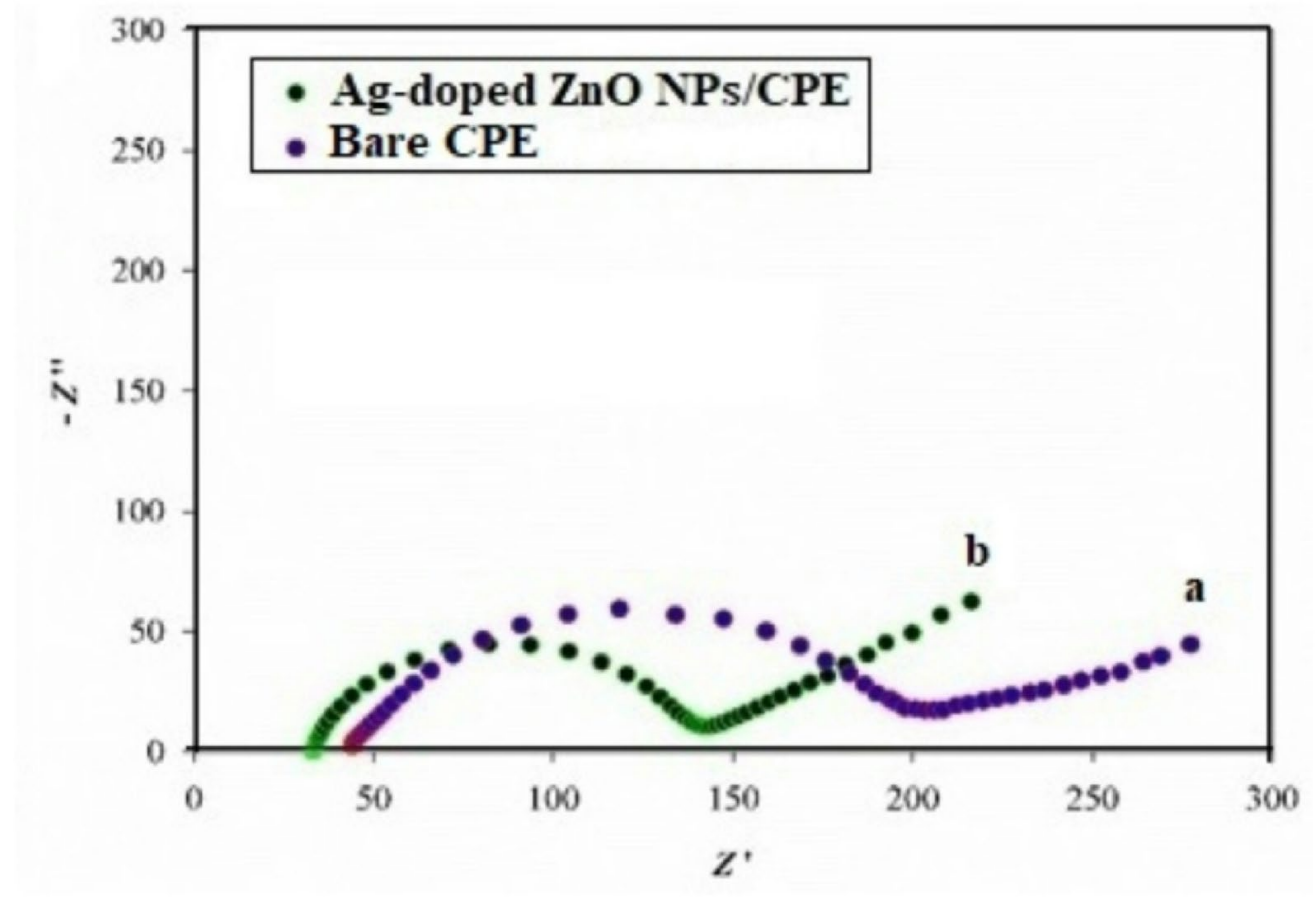
Nyquist plots of bare CPE **a** and Ag-doped electrode **b** using 10 mmol/L (K_4_[Fe(CN)_6_]) / (K_3_[Fe(CN)_6_]) redox couple in 0.1 mol/L KCl solution

The electrocatalytic performance of the synthesized Ag-doped ZnO NPs modified CPE was assessed using CV and EIS techniques [43]. The electrochemical behavior of 10 mmol/L (K_4_[Fe(CN)_6_]) / (K_3_[Fe(CN)_6_]) redox couple, in 0.1 mol/L KCl electrolyte solution [44], was investigated at both bare CPE and Ag-doped ZnO modified electrodes. As shown in Fig. 3, the Ag-doped modified electrode exhibited an enhanced voltammetric response compared to the unmodified one. To determine the electroactive surface area of both electrodes, CV voltammograms of the redox couple were recorded, and the electroactive surface area was calculated to be 0.91 cm^2^ for the Ag-doped modified electrode, and 0.67 cm^2^ for the unmodified electrode, confirming that Ag-doping effectively increased the electroactive surface area and consequently enhanced electrode sensitivity. Moreover, EIS analysis was carried out to demonstrate the charge transfer kinetics and to further validate the modification effect. The Nyquist plots (Fig. 4) revealed that Ag-doped ZnO NPs modified CPE had a semi-circle with a smaller diameter than that of the unmodified electrode. Correspondingly, the charge transfer resistance (R_ct_) decreased from 143 Ω (unmodified) to 100 Ω (Ag-doped), indicating facilitated electron transfer. The double layer capacitance (C_dl_) was calculated for Ag-doped electrode as 2.53 × 10^− 6^ F compared to 2.83 × 10^− 6^ F for the unmodified one. These findings confirm successful modification, together with enhanced charge transfer kinetics and electrocatalytic activity at the Ag-doped ZnO NPs modified CPE surface.

### Optimization of Ag-ZnO percent, analytical and electrochemical parameters

Various experimental variables play a crucial role in redox systems. They were investigated through monitoring the electrochemical behavior of 2.45 mmol/L CLZ on the surface of Ag-doped ZnO NPs modified CPE.

#### Effect of Ag-ZnO ratio

**Fig. 5:**
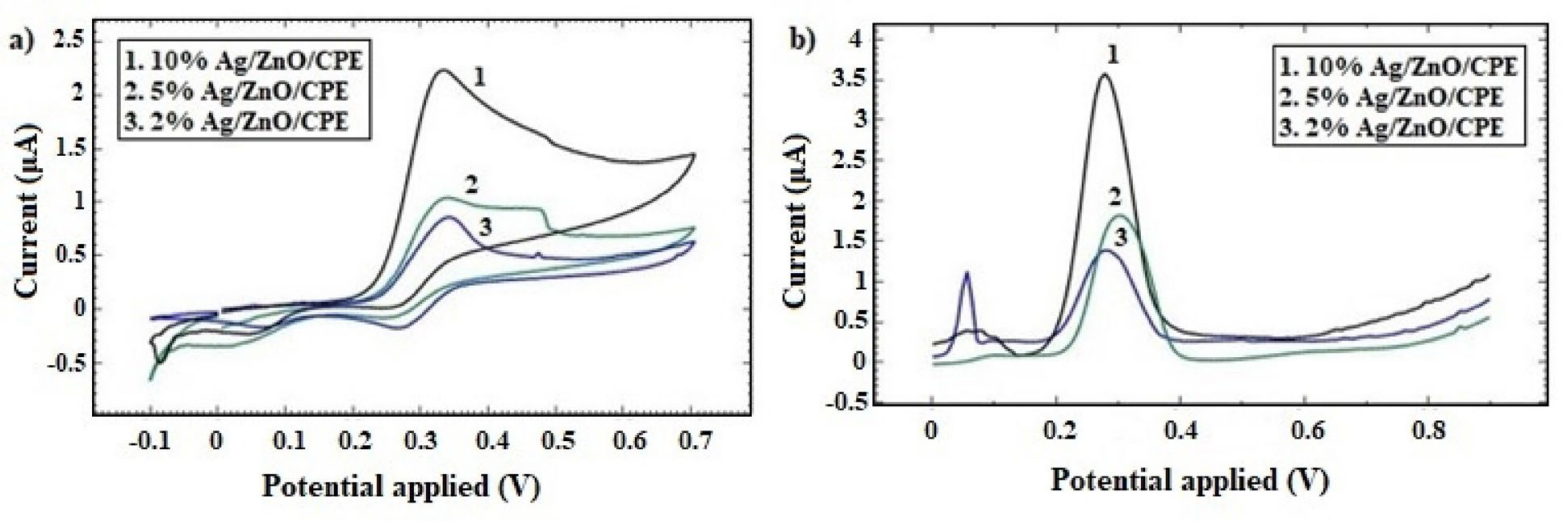
CV **a** and DPV **b** voltammograms of 2.45 mmol/L CLZ in KCl solution using modified CPE with various amounts Ag-ZnO NPs

The proper amount of the selected modifier was determined by recording CLZ voltammetric behavior on the surface of modified CPE. Three different ratios of the modifier Ag-doped ZnO NPs and graphite powder were employed; 2, 5, and 10% w/w. The modifier amount of 10% improved the anodic peak in both CV and DPV techniques (Fig. 5a-b). It almost doubled the I_p_ value obtained with other amounts. Therefore, 10% Ag-doped ZnO NPs was realized as the optimum choice for CPE modification, due to the acquired significant elevated sensitivity.

#### Effect of pH on sensor’s performance

**Fig. 6:**
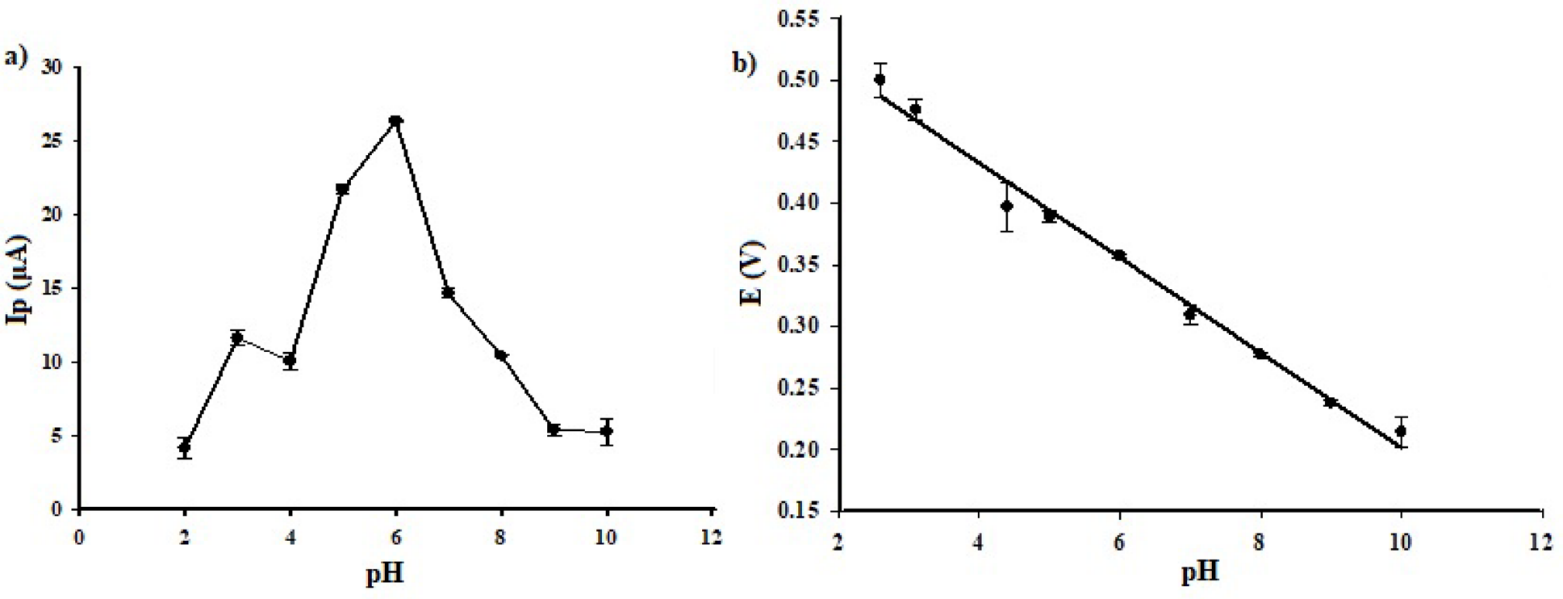
Effect of pH (using BRB) on I_p_ **a** and E_p_ **b** of 2.45 mmol/L CLZ on Ag-doped ZnO NPs/CPE

DPV technique was held over a pH range of 2.0 to 10.0 using BRB solution. To reach the optimum effectiveness, a buffer concentration of 0.04 mol/L was employed; based on previously published studies on similar applications [20, 45, 46]. As shown in Fig. 6-a, the obtained I_p_ were noticeably enhanced upon increasing the pH from 2.0 to 6.0. The highest value was recorded at pH 6.0. Above pH 6.0, a dramatic decrease in I_p_ was observed. pH 6.0 is suggested to facilitate the oxidation of CLZ. Thus, it was selected as the optimum pH for further optimization of the remaining parameters. Furthermore, a plot that relates the variously adopted pH values to the corresponding peak potentials (*E*_p_) was constructed (Fig. 6-b). E_p_ were shifted towards more negative potential values when pH values were increased. This deducts that the redox process of CLZ is proton dependent. The obtained regression equation is $\:E\:\left(V\right)=0.5686-0.0363\:pH$, with r^2^ of 0.988. According to Nernstian relation “$\:m=0.059\:x\:\frac{h}{n}$*”*, where; *m* is slope and *(h/n)* is ratio of protons to electrons, the obtained slope of -0.0363 V/pH value, was close to the theoretical Nernstian value of -0.0295 V/pH. This suggests that CLZ oxidation process at the proposed electrode involved a proton to electron ratio of 1: 2. This finding is consistent with the previously published mechanism for CLZ oxidation [20, 47].

#### Effect of scan rate

**Fig. 7:**
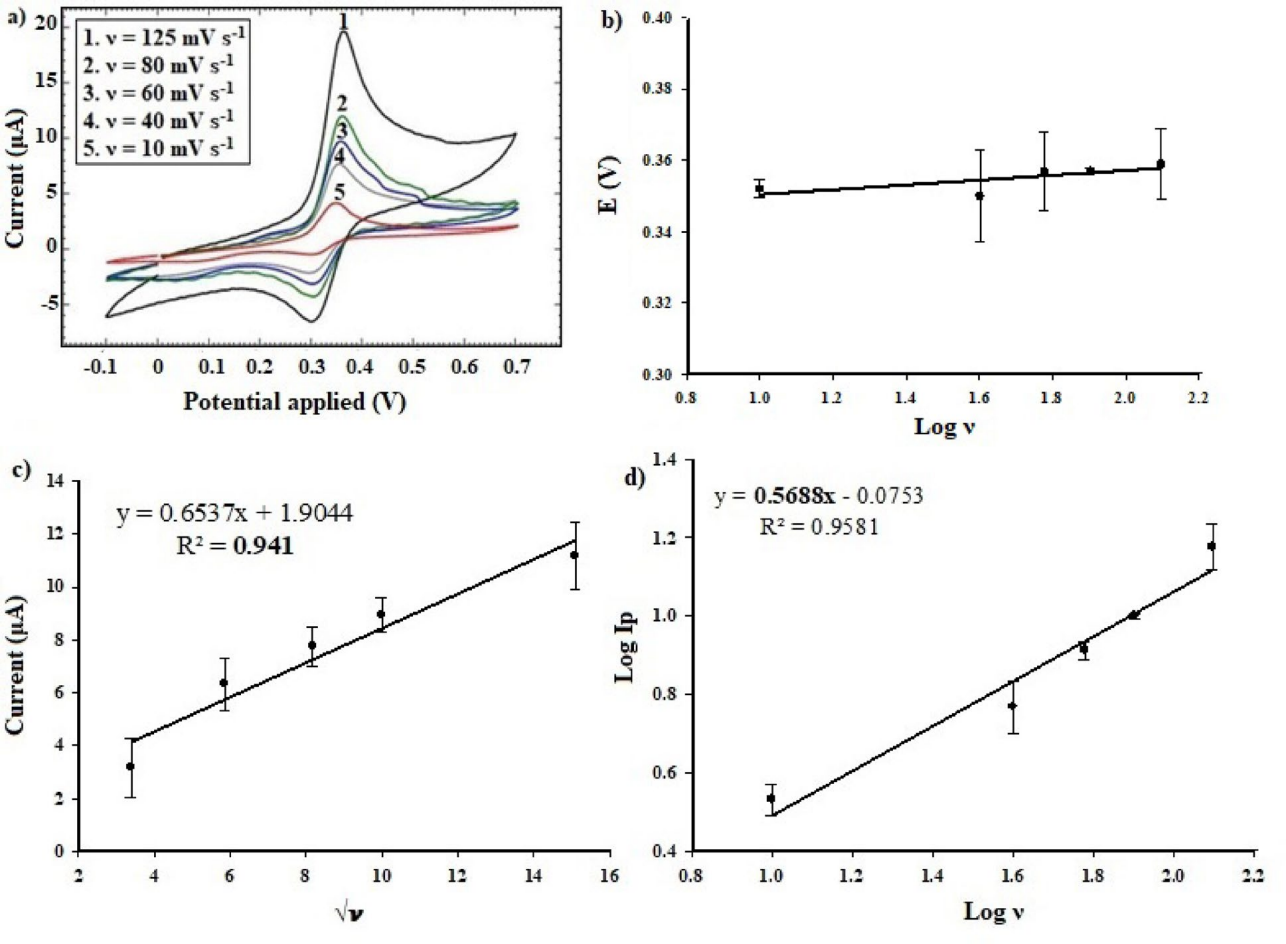
**a** CV voltammograms of 2.45 mmol/L CLZ at different scan rates (10–125 mV s^− 1^) on Ag-doped ZnO NPs modified CPE, **b** relation between oxidation E_p_ vs. log ν, **c** shows correlation between current and square root of ν, and **d** correlates log values of I_p_ and ν

Scan rate (ν) is an influential factor that reveals the nature and response of the adopted electrode towards the electrochemical behavior of assayed molecules. This declares whether the electrochemical process has a reversible nature or not, and whether it is controlled by adsorption or diffusion. CV technique was applied over ν ranged from 10 to 125 mV s^− 1^. As shown in Fig. 7-a, the recorded anodic I_p_ were increasing with increased ν values. Figure 7-b, shows E_p_ values that remained almost constant with increased log ν values, indicating the E_p_ is independent on ν and declaring a reversible mechanism. Also, charge transfer coefficient (α) was estimated from the obtained slope *(*$\:\frac{2.3\:RT}{\left(1-\:\alpha\:\right)nF}$*)*, by Laviron’s method, where, *R* is the gas constant (8.314 J/mol.K), *T* is the temperature (289 K), *n* is the number of electrons (2) and *F* is the Faraday constant (96485 C/mol) [48]. The calculated α value of 3.13 falls outside the acceptable range (0–1) [49], further supporting the unlikelihood of an irreversible reaction, and indicating the inapplicability of Laviron’s method for estimating the kinetics of the proposed reaction. So that, α was alternatively calculated by the equation **“**$\:\varvec{\alpha\:}=\:\frac{47.7}{\varvec{n}(\varvec{E}\varvec{p}-\varvec{E}\varvec{p}/2)}$**”** [35, 50], where, ***Ep/2*** is the half peak potential. The obtained α value of 0.51 suggests symmetric transition state and electrochemical equilibrium between reactants and products [51]. These findings prove the reversible nature as well as fast electron transfer during the oxidation process of CLZ on Ag-doped ZnO NPs modified CPE. As a further investigation, I_p_ was correlated to square root of ν (Fig. 7-c). The obtained regression equation is $\:Ip=0.6537\:\sqrt{\nu\:}+1.9044$, with r^2^ of 0.941; indicating linearity and reversibility. On the other hand, log I_p_ was correlated to log ν as “$\:Log\:Ip=0.5688\:log\:\nu\:-0.0753$” (Fig. 7-d). Normally, the slope of 0.5 indicates the diffusion controlled, 1.0 indicates the adsorption controlled process [52]. Thus, the oxidation of CLZ at the surface of Ag-doped ZnO NPs modified CPE is suggested to follow a mixed diffusion-adsorption mechanism, with diffusion as the predominant controlling process, accompanied by fast charge transfer.

#### Adjustment of DPV input parameters

**Fig. 8:**
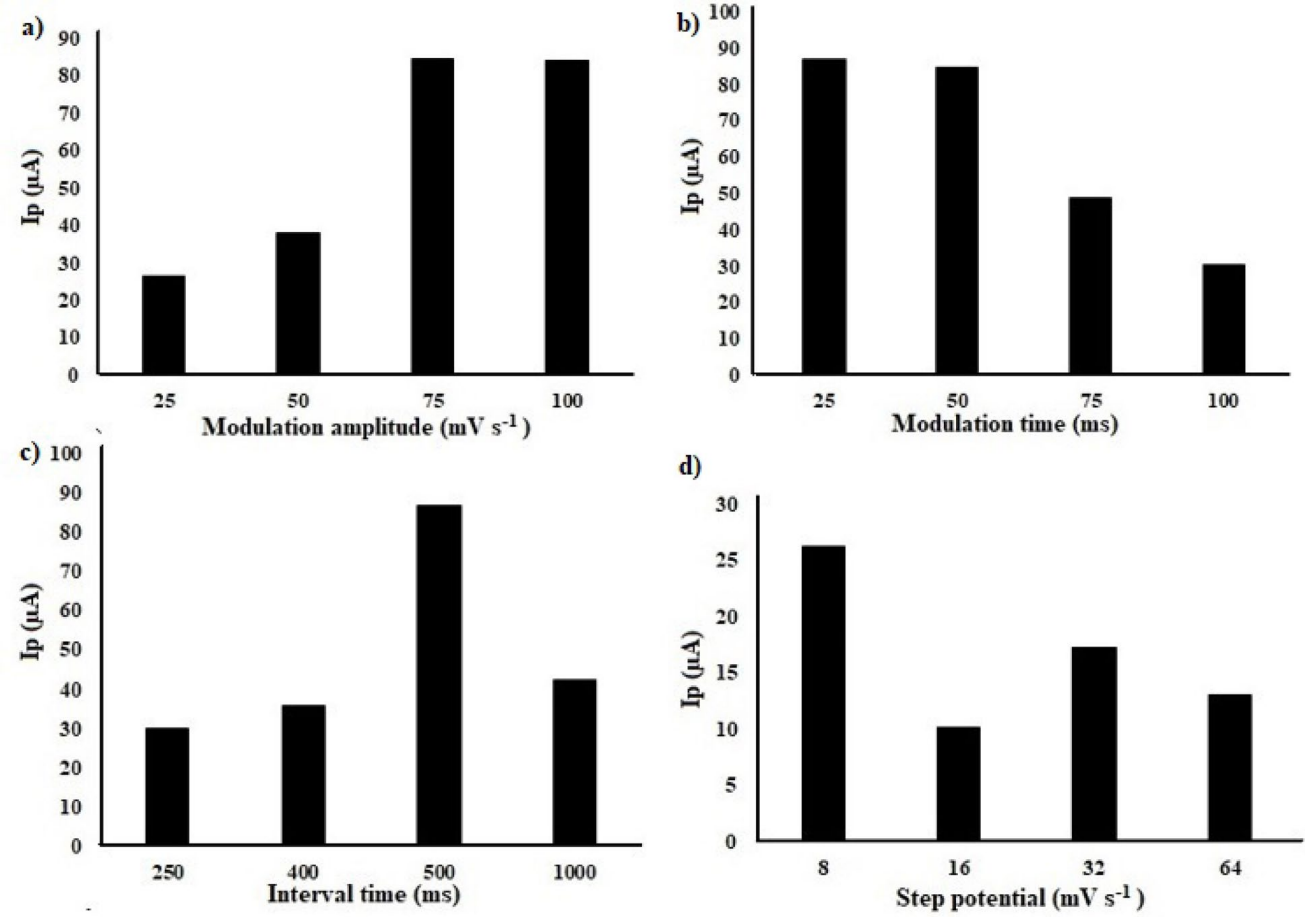
Effect of DPV input parameters: **a** Modulation amplitude, **b** Modulation time, **c** Interval time and **d** step potential, on 2.45 mmol/L CLZ (pH 6), at Ag-doped ZnO NPs modified CPE

The operational parameters of DPV technique significantly impact the sensitivity and shape of the obtained peaks [34]. Regarding the proposed method, the optimum DPV inputs were carefully evaluated. Examinations were conducted for modulation amplitude over the range of 25 to 100 mV s^− 1^, modulation time and interval time values ranging from 25 to 100 ms and 250 to 1000 ms for both factors, respectively, and step potential that fluctuated from 8.0 to 64 mV s^− 1^. As illustrated by Fig. 8, the optimal electrochemical responses, of CLZ out of DPV technique, were obtained at 75 mV s^− 1^, 25 ms, 500 ms and 8.0 mV s^− 1^ for modulation amplitude, modulation time, interval time and step potential, respectively.

### Electrochemical behavior of CLZ on the modified CPE

CV was employed to study the electrochemical behavior and the potential mechanism of CLZ redox process, at pH 6.0, on the surface of the developed Ag-doped ZnO NPs modified CPE. CV responses were recorded over a scanning potential of -100 to 700 mV. Both anodic and cathodic peaks appeared at 327 and 272 mV, respectively. CLZ had a sharper and more well-defined anodic peak compared to the cathodic one. This suggests that the product, produced through the oxidation reaction, is partially reversible through the reverse scan.

### Electrochemical sensor validation

The reliability of the proposed DPV method together with the dependability of the developed Ag-doped ZnO NPs modified CPE sensor in assaying CLZ in human saliva samples were accredited in agreement with FDA guidelines for validation of bioanalytical methods [40]. Spiked saliva samples were used due to challenges in engaging resistant schizophrenic patients with co-morbid SUD who are prescribed CLZ. Mainly, due to their unwillingness to admit illicit substance use, together with their non-adherence to the prescribed CLZ regimen. However, saliva collection and spiking protocols were carefully adopted to accurately reflect realistic CLZ levels.

#### Calibration curve, lower limit of quantitation (LLOQ) and sensitivity

**Fig. 9:**
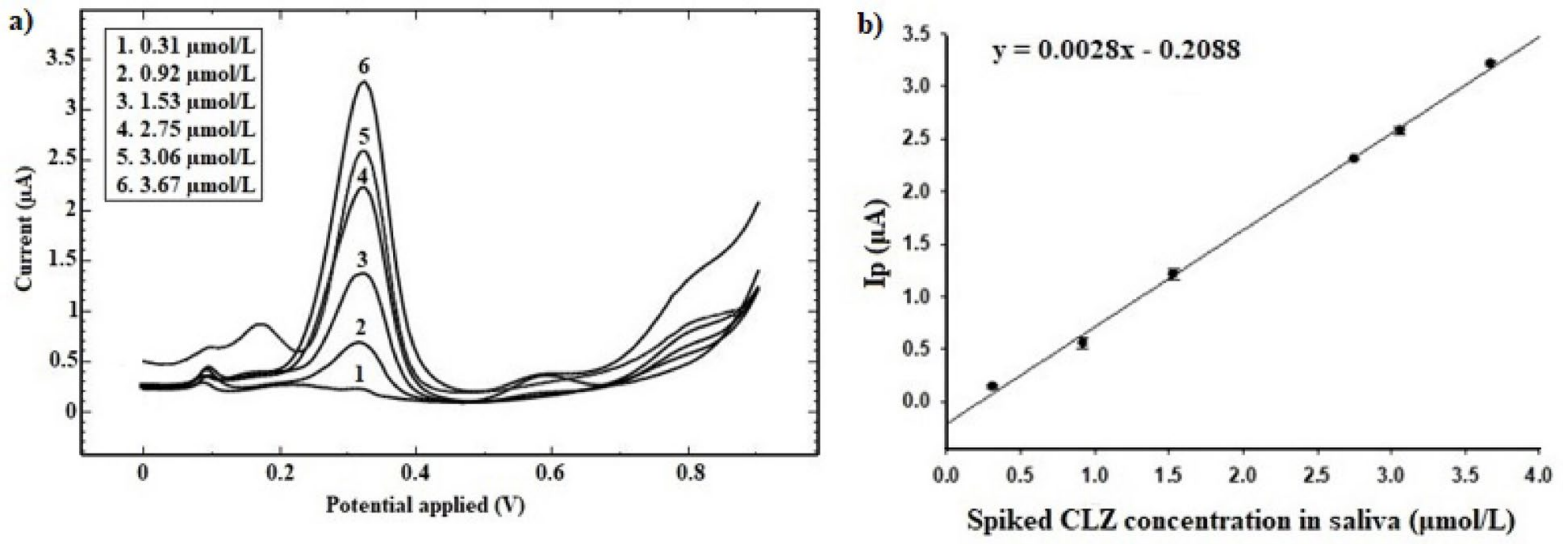
**a** DPV voltammograms of CLZ calibrators in saliva and **b** Corresponding calibration curve using the developed Ag-doped ZnO NPs modified CPE sensor for CLZ determination

Six non-zero calibrators of CLZ (0.31, 0.92, 1.53, 2.75, 3.06 and 3.67 µmol/L CLZ in saliva), were assayed using the proposed method, and the DPV voltammograms were recorded as shown in Fig. 9-a. These were involved to construct the calibration curve (Fig. 9-b). I_p_ values were plotted against the corresponding concentrations. The least squares method was applied on the calibration data to calculate the linearity parameters; correlation coefficient, slope (b), intercept (a), standard deviation of slope (S_b_), standard deviation of intercept (S_a_), F experimental and F critical. All calculated parameters lied within the acceptable ranges (Table 1). The proposed method showed excellent linearity with regard to the obtained high correlation coefficient (r ˃ 0.99), together with low standard deviation of slope (%S_b_ ˂ 2%) and high experimental F with small critical F values. This indicated good regression model with low residuals scattered around the regression line. To further ensure the reliability of the proposed method; back calculations of non-zero calibrators’ concentrations were carried out. They were found within the acceptable ranges (± 20% for LQC, MQC and HQC and ± 25% for LLOQ and ULOQ).

The LLOQ was 0.31 µmol/L CLZ in saliva. It is lower than the expected CLZ levels in saliva (1.56 µmol/L for patients on recent CLZ regimen and 2.81 µmol/L for those adhered to CLZ therapy for more than two years [30]). This assured the suitability of the proposed method in assaying real CLZ containing saliva samples. Moreover, the sensitivity of the proposed method was confirmed by conducting four determinations at LLOQ level (0.31 µmol/L CLZ in saliva). Calculated recovery%, RSD% and total error were found to be 118.56% (± 25%), 4.57% (± 25%) and 23.14% (˂ 40%). Thus, these results approved the sensitivity of the proposed method in assaying trace levels of CLZ in saliva.

**Table 1:** Regression parameters of the proposed DPV method for CLZ determination in saliva using Ag-doped ZnO NPs/CPE sensor

Parameter	CLZ
Linearity range (µmol/L saliva)	0.31–3.67
LLOQ (µmol/L saliva)	0.31
Correlation coefficient (r)	0.9990
Slope ± S_b_	0.0028 ± 6.22 × 10^− 5^
Intercept ± S_a_	-0.2088 ± 0.0482
S_b_%	0.0221
S _y/x_^*^	0.0490
F experimental	2052
F critical	1.42 × 10^− 6^

*S _y/x_ refers to standard deviation of residuals

#### Accuracy

Intra-day together with inter-day accuracy of the proposed DPV method were assessed. The aforementioned procedure was applied at QC levels of 0.31, 0.92, 1.53, 2.75 and 3.67 µmol/L spiked CLZ in saliva. Each QC level was determined four times. Then, recovery%, RSD% and total error % *(Bias % + RSD %)* were calculated. As per Table 2, the values of recovery% and RSD% were all within the acceptable limits of ± 20% (± 25% for LLOQ and ULOQ levels). In addition, values of total error were all within ± 30% (± 40% for LLOQ and ULOQ levels). These findings highlight the consistency of the sensor’s response across replicates. So that, the proposed method proved distinct accuracy and precision that guarantee the reliability of assaying CLZ in saliva samples.

**Table 2:** Accuracy of the proposed DPV method for CLZ determination in saliva using Ag-doped ZnO NPs/CPE sensor (*n* = 4)

Spiked CLZ concentration*	Intra-day			Inter-day		
	Recovery %	RSD %	Total error %	Recovery %	RSD %	Total error %
0.31	116.84	4.47	21.31	125.10	4.34	29.44
0.92	91.92	6.73	16.01	87.55	7.43	28.11
1.53	98.21	3.19	4.69	112.36	13.11	25.46
2.75	98.67	2.48	2.62	95.58	6.97	10.26
3.67	99.88	3.64	3.52	104.71	3.97	8.68

*Concentration in µmol/L saliva

#### Selectivity and specificity

Regarding the method’s selectivity, blank saliva samples from ten individual sources were analyzed by DPV. The responses obtained from 80% of the un-spiked samples were below the LLOQ level of CLZ. Thus, the developed method had successfully fulfilled the acceptance criteria that is ≥ 80% of un-spiked samples should be below LLOQ (BQL). Furthermore, LLOQ and HQL levels of spiked saliva samples were analyzed in triplicates by DPV. The obtained results of recovery% were within ± 25% for LLOQ (124.67%) and ± 20% for HQL (108.29%). Accordingly, the developed DPV method proved its ability to selectively quantify CLZ in saliva samples with no potential interferences from endogenous saliva matrix.

**Fig. 10:**
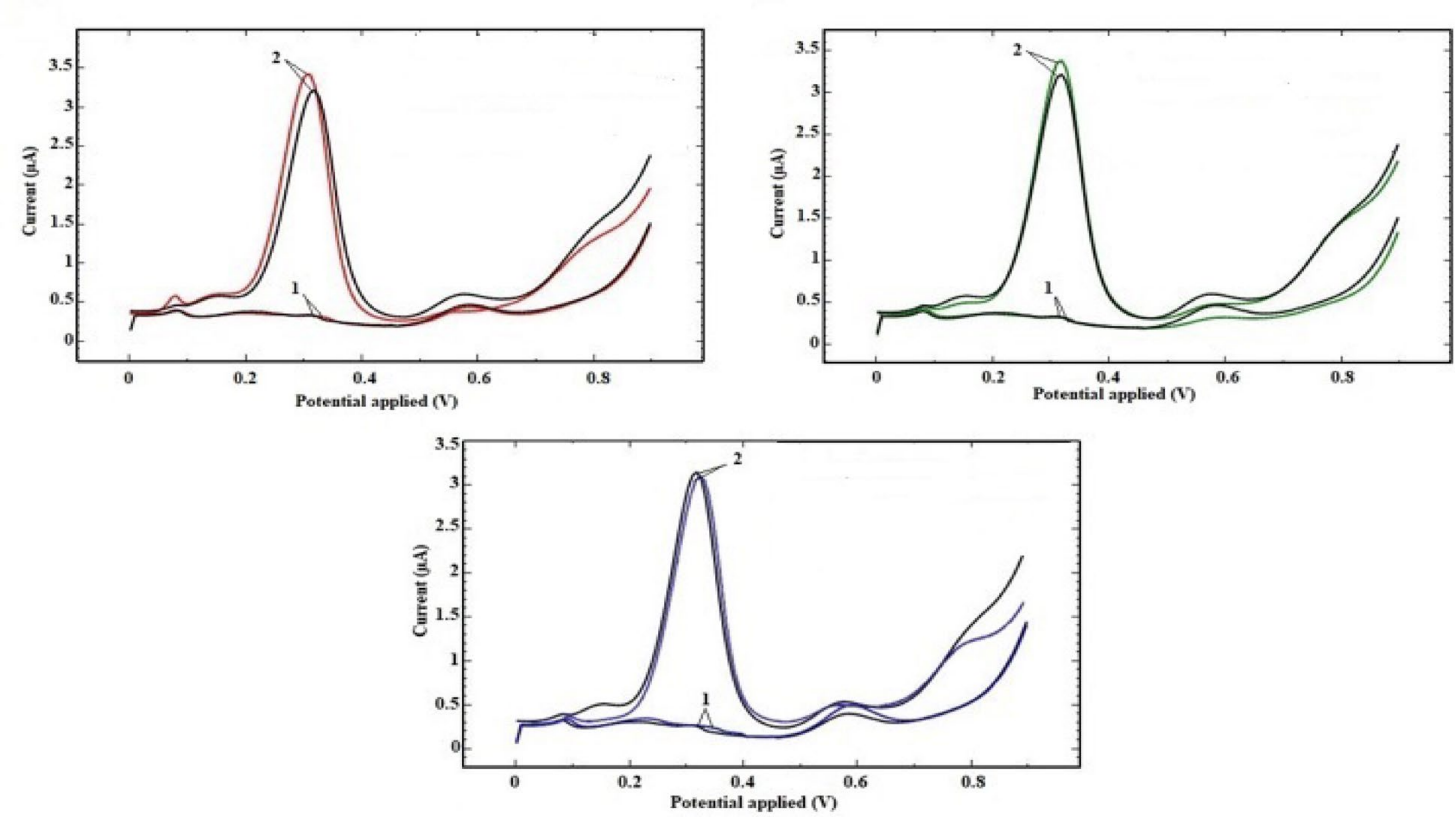
DPV voltammograms of CLZ ( ) and its binary mixtures with BAC ( ), IBF ( ), and PC ( ) at LLOQ (1) and ULOQ (2)

Moreover, the specificity of the proposed DPV method towards CLZ in presence of some co-administered medications was evaluated. Saliva samples were spiked with CLZ at LLOQ and ULOQ levels (0.31 and 3.67 µmol/L saliva, respectively), in addition to some of the potentially interfering drugs; baclofen (BAC), ibuprofen (IBF) and paracetamol (PC) at concentrations of 2.34, 2.42 and 3.31 µmol/L saliva, respectively. Then, triplicate analysis was carried out by DPV method using Ag-doped ZnO NPs modified CPE. DPV voltammograms (Fig. 10) obtained for CLZ, were similar to those recorded when same CLZ concentrations were assayed in mixtures with the interfering drugs. In all cases, only CLZ corresponding peak at 327 mV was observed, while no additional peaks indicating interference were detected. Thus, it can be anticipated that the co-administered drugs had negligible effect on CLZ determination in saliva. As a conclusion, the developed DPV method together with the utilized sensor showed noticeable selectivity and specificity to CLZ even with the presence of potential exogenous interferents in the assayed.

samples.

#### Stability

The stability of CLZ in saliva matrix was evaluated on various stability aspects. Bench-top stability was performed on QCs samples that have been left at room temperature for six hours before analysis. On the other hand, freeze-thaw stability tests were performed on QCs samples after exposing them to three cycles of freeze and thaw, with at least 12-hours’ time interval between cycles. In addition, long term stability involved analysis of QCs samples after storing them at -20 °C for 30 days. While, the stability of CLZ stock solution was examined after 6.0 days of storing it at 25 °C and also after 30 days of storage at 4.0 °C. The aforementioned stability examinations were held by the proposed DPV method at LQC and HQL levels of CLZ in saliva (0.92 and 2.75 µmol/L saliva, respectively); each in triplicate. Results obtained from QCs stability samples were compared to those obtained from freshly prepared QCs (Table 3). The calculated recovery% values for both QC levels were within ± 20%. This approved the outstanding stability of CLZ in saliva matrix throughout whole phases that are involved in the CLZ assay process.

**Table 3:** Evaluation of CLZ stability in saliva by applying the developed DPV method using Ag-doped ZnO NPs/CPE sensor (*n* = 3)

Stability approach	Recovery%	
	0.92 µmol/L CLZ in saliva	2.75 µmol/L CLZ in saliva
Bench-top	100.14	101.22
Freeze-thaw	105.33	109.98
Long term	90.53	95.36
Stock		
4.0 °C	85.22	91.53
25 °C	111.37	114.79

#### Inter-electrode variability

As an additional study, robustness of the proposed Ag-doped ZnO NPs based CPE sensor was investigated through batch-to-batch reproducibility. Under identical conditions; five sensors were prepared independently and utilized for determination of CLZ at LLOQ, MQC and ULOQ levels, (0.31, 1.53 and 3.67 µmol/L saliva, respectively). Each QC level was assayed in triplicates using the proposed method. The I_p_ responses obtained from different sensors showed RSD% of 4.09%, 3.73% and 2.96% for LLOQ, MQC and ULOQ, respectively. The RSD% values were all below 5%, indicating satisfactory batch-to-batch reproducibility. So that, sensor’s robustness and ease of fabrication are confirmed.

Referring to previously reported electrochemical methods for CLZ assay (Table 4); the developed method had comparable and even higher potential to be adopted for routine TDM of CLZ. This is referred to its ability to assay CLZ levels in human saliva, with reliable analytical performance in terms of linear range and sensitivity (i.e. LLOQ). Moreover, it depends on a facile and economic method for the synthesis of the utilized electrochemical sensor. Saliva, as a biological matrix, offers various advantages over other traditional matrices like plasma and urine. Its non-invasive collection makes it a patient friendly option. In addition, it omits the need for trained personnel and sophisticated instruments. As well as, no sample treatment is needed prior to analysis. All of these make it more convenient for frequent CLZ monitoring. Furthermore, it accurately reflects CLZ levels throughout its therapeutic range, thus it enables the establishment of a superior tool for TDM of CLZ compared to other techniques.

**Table 4:** Comparison of the electrochemical sensors and methods for CLZ determination

Electrochemical sensor	Synthesis method	Electrochemical technique	Bioanalytical application Application matrix	Linearity range (µmol/L)	LLOQ/LOD (µmol/L)	Refs.
AgTiO_2_-CPE^a^	Liquid impregnation	Square wave voltammetry	Not applied Buffered solution	0.90–40	0.43 * 10^−3^	[35]
EPGCE^b^	Commercially available	Adsorptive stripping voltammetry	Applied Urine Plasma	46–122 76–153	12 24	[53]
CNT-Chitosan composite film/gold electrode^c^	Electrodeposition & casting methods	Differential pulse voltammetry	Applied Serum	1.0–3.0	0.50	[54]
VMSF/p-SPCE^d^	Electrochemical polarization & electrodeposition	Differential pulse voltammetry	Applied Whole blood	0.50–12	0.11	[23]
Catechol-Chitosan redox cycling modified LOC	Electrodeposition	Cyclic voltammetry	Applied Serum	0.49–25	2.45	[25]
r-GO-modified microelectrode^e^	Electrodeposition	Cyclic voltammetry	Applied Serum Whole blood	0.30–5.0 0.30–5.0	0.54 0.64	[55]
Ag-doped ZnO NPs/CPE	Co-precipitation	Differential pulse voltammetry	Applied Saliva	0.31–3.67	0.31	This work

^a^ Silver doped TiO2 modified carbon paste electrode

^b^ Electrochemically pretreated glassy carbon electrode

^c^ Multi-walled carbon nanotubes-chitosan composite film coated on gold electrode

^d^ Vertically ordered mesoporous silica nano-membrane film on the electrochemically polarized screen printed electrode

^e^ Reduced graphene oxide modified microelectrode

## Conclusion

The current work presents the first electrochemical sensor (Ag-doped ZnO NPs/CPE), designed for TDM of CLZ in human saliva using spiked samples. The sensor was synthesized by a simple and economic co-precipitation method, thus, its facile and high quality reproduction was ensured. The developed electrochemical method (DPV) showed excellent sensitivity, that enables a reliable determination of CLZ within its therapeutic range. In addition, it exhibited high accuracy and precision for CLZ quantification in saliva, offering a patient-friendly route for TDM. Consequently, the proposed sensor provides a reliable and accurate approach for regular and non-invasive TDM of CLZ. Furthermore, it established a foundation for future investigations regarding the potentiality of employing the proposed sensor as a point-of-care testing device (POCT).

## Data Availability

All data generated or analyzed during this study are included in this published article.

## Abbreviations

BRB: Britton-Robinson buffer
CLZ: Clozapine
CPE: Carbon paste electrode
CV: Cyclic voltammetry
DPV: Differential pulse voltammetry
EDX: Energy dispersive X-ray spectroscopy
EIS: Electrochemical impedance spectroscopy
FTIR: Fourier-transform infrared spectroscopy
HQC: High quality control
LLOQ: Lower limit of quantitation
LQC: Low quality control
MQC: Mid quality control
NPs: Nanoparticles
PEG: Polyethylene glycol
POCT: Point-of-care test
QCs: Quality controls
SUD: Substance use disorder
ULOQ: Upper limit of quantitation
